# Free Energy Landscape and Multiple Folding Pathways of an H-Type RNA Pseudoknot

**DOI:** 10.1371/journal.pone.0129089

**Published:** 2015-06-01

**Authors:** Yunqiang Bian, Jian Zhang, Jun Wang, Jihua Wang, Wei Wang

**Affiliations:** 1 Collaborative Innovation Center of Advanced Microstructures and Department of Physics, Nanjing University, Nanjing 210093, China; 2 Shandong Provincial Key Laboratory of Functional Macromolecular Biophysics, Institute of Biophysics, Dezhou University, Dezhou 253023, China; University of Leeds, UNITED KINGDOM

## Abstract

How RNA sequences fold to specific tertiary structures is one of the key problems for understanding their dynamics and functions. Here, we study the folding process of an H-type RNA pseudoknot by performing a large-scale all-atom MD simulation and bias-exchange metadynamics. The folding free energy landscapes are obtained and several folding intermediates are identified. It is suggested that the folding occurs via multiple mechanisms, including a step-wise mechanism starting either from the first helix or the second, and a cooperative mechanism with both helices forming simultaneously. Despite of the multiple mechanism nature, the ensemble folding kinetics estimated from a Markov state model is single-exponential. It is also found that the correlation between folding and binding of metal ions is significant, and the bound ions mediate long-range interactions in the intermediate structures. Non-native interactions are found to be dominant in the unfolded state and also present in some intermediates, possibly hinder the folding process of the RNA.

## Introduction

As a major type of macromolecule essential for life, RNAs carry out numerous biological functions including translating genetic information into proteins, regulating gene expression, catalyzing biochemical process, etc. For a better understanding of their functions, knowledge of how they achieve functional structures through folding is necessary. Furthermore, comparison of the folding mechanisms of two distinct biopolymers, RNA and proteins, may reveal different physical-chemical interactions governing folding and deepen our understanding of the structure formation process of biomolecules.

RNA pseudoknots are examples of minimal structural motifs in RNA with tertiary interactions. They have been found play important roles in self-splicing, stimulating ribosomal frameshifting, forming the catalytic core, etc [1, 2]. In addition to their functional importance, RNA pseudoknots also provide excellent models for studying the folding mechanism of RNAs. This is because they contain many types of interactions commonly seen in RNAs, including canonical and non-canonical base pairs, tertiary interactions such as the A-minor interactions often seen in loop-stem triplex, coaxial stacking, and particularly the metal ion-nucleotides interactions. Moreover, the study of pseudoknots can also be beneficial for developing new approaches for RNA structure prediction, since the non-nested topology, non-canonical interactions and loop entropy of pseudoknots cause significant difficulties in developing efficient sampling algorithms, as well as in determining energy rules[3–8].

There have been lots of experimental and theoretical works studying the folding and unfolding mechanism of RNA pseudoknots [9–21], conformational switch between metastable structures [22–24], roles of metal ions on structure stability and folding process [25–30], etc. For instance, in an early study, Draper and colleagues investigated the effects of mono and divalent ions in the folding process of an mRNA pseudoknot and discussed different effects of metal ions of different size and valence [15]. Chen et al. studied the mechanical folding and unfolding of a pseudoknot in human telomerase RNA (hTR) by optical tweezers and discovered a stepwise folding mechanism as well as a one-step unfolding mechanism [9]. They also detected the existence of nonnative intermediates and provided evidence that the folding of both hairpin and pseudoknot takes complex pathways. Also using optical tweezers, Green et al. studied the frameshifting pseudoknot from infectious bronchitis virus (IBV) and its mutants, giving their thermodynamics and kinetics as a function of forces, and also their dependence on Mg^2+^ ions [25]. Wu et al. studied the operator of the rpsO gene transcript and discovered that it interchanges spontaneously between a pseudoknot conformation and a double-hairpin conformation[22]. Theoretically, in an early all-atom molecular dynamics (MD) simulation of a pseudoknot from beet western yellow virus, Csaszar et al. revealed several early unfolding events at an elevated temperature (400 K) [20]. Chen's group developed a series of coarse-grained RNA models, which enabled them to predict the conformational entropy, loop-helix tertiary contacts, as well as structures of loops [10–13]. Cho and colleagues performed systemic simulations on different pseudoknots based on a coarse-grained model [14]. They found that the folding landscapes of RNAs with a similar topology are significant sequence-dependence. Their work also showed that the folding mechanism of pseudoknot is mostly determined by the relative stabilities of secondary structures. With all-atom simulations, we studied the thermal unfolding kinetics of an RNA pseudoknot within gene32 mRNA of bacteriophage T2 [19]. We detected multiple intermediates and found that the transition states are rather diverse, with a conclusion that the unfolding of the specific RNA follows multiple pathways.

**Fig 1:**
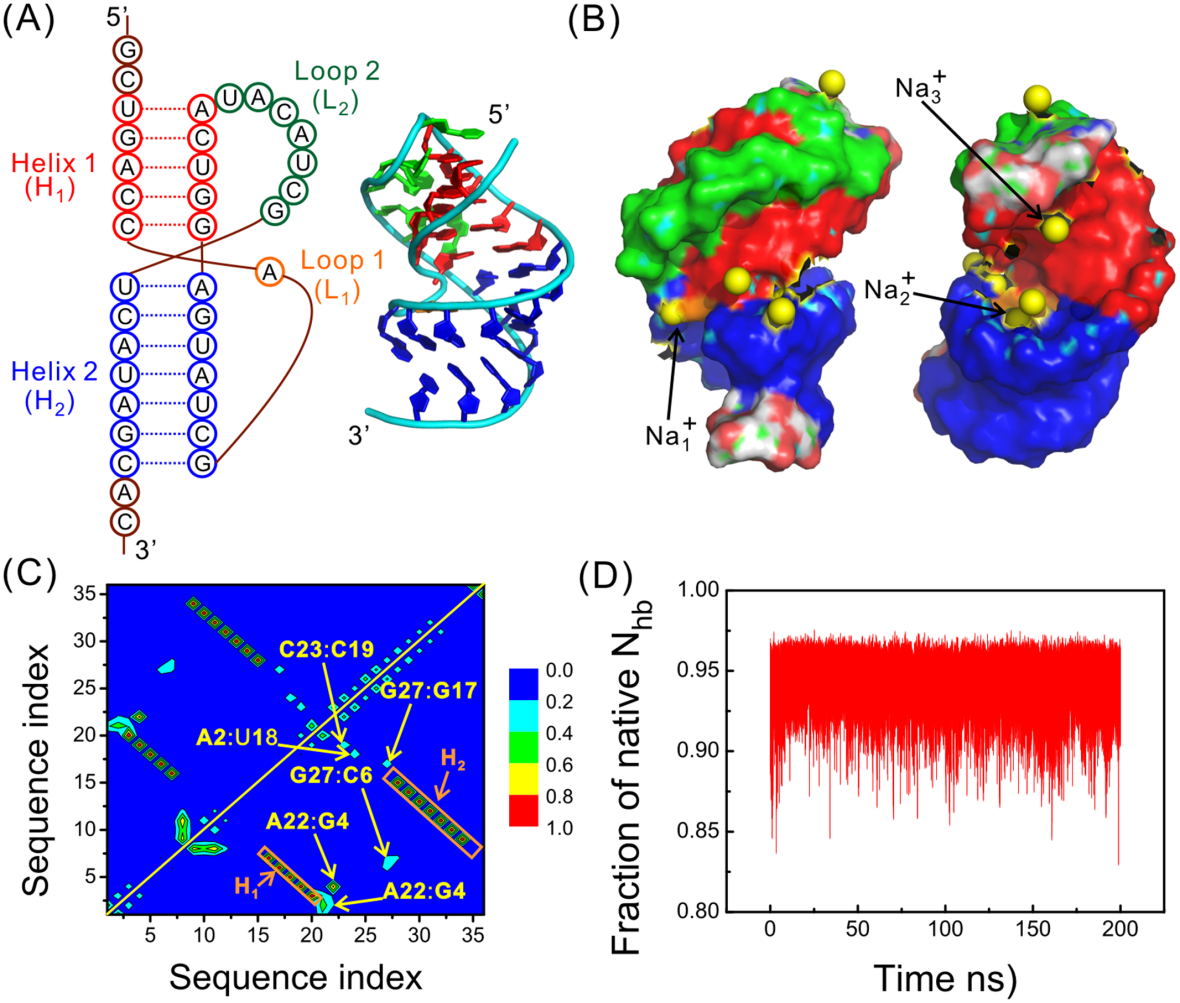
Native structure of the RNA pseudoknot within gene32 mRNA of bacteriophage T2. (A) Secondary and tertiary structures of the RNA pseudoknot (PDB code: 2TPK). Two helices are labeled as Helix 1 (H_1_) and Helix 2 (H_2_) and are colored red and blue, respectively. Two loops are labeled as Loop 1 (L_1_) and Loop 2 (L_2_), and are colored orange and green, respectively. The same color code is used in all the figures unless otherwise indicated. (B) Surface model of a typical structure, viewed from two perpendicular directions, taken from a 200 ns MD simulation started from the native structure. Cations are plotted as yellow spheres. (C) Hydrogen bond (HB) map averaged over all the conformations in the same MD run. The formation probabilities are indicated by different colors, as quantified by the color scale beside the figure. The red labels indicate the HBs within two helices, and the yellow ones indicate that between H_2_ and L_2_, i.e., the tertiary interactions. (D) The fraction of the formed native HBs as a function of time in the same MD run.

Although there are many excellent experimental as well as theoretical works, the atomistic scenario of the folding process of RNA pseudoknots is still lacking. All-atom molecular dynamics simulations can be of help. However, such studies are still rare for RNA pseudoknots, mostly due to the slow kinetics of the molecules and the resulted difficulty in achieving enough sampling in the relevant phase space [18]. Here we study the folding free energy landscape (FEL) of the pseudoknot within the gene32 mRNA of bacteriophage T2 (Fig 1A and 1B), with both RNA and water molecules explicit modeled. To accelerate the sampling rate in phase space, we adopt an advanced sampling technique named bias-exchange metadynamics (BEMD) [31]. This powerful technique enables us to explore the free energy landscape and identify potential folding intermediates. The FEL, combined with the kinetic results for the same RNA obtained in our previous work [19], provides a comprehensive view of the folding process. Based on the simulation results we propose an atomistic scenario for the folding process and discuss the relevance of our results with previous experimental and theoretical findings. We also discuss the roles played by native and non-native interactions and metal ions in the folding process.

## Materials and Methods

### Preparation of the system

The native structure of the RNA pseudoknot (PDB code: 2TPK) was solvated in a periodic box with 12525 TIP3P water molecules. Na^+^ and Cl^-^ ions were added to neutralize the system and to maintain a salt concentration of 100mM. The simulations were performed with Gromacs (version 4.5.5) [32] and force field amber99sb_parmbsc0 [33], which combined the amber99sb force field with the parmbsc0 nucleic acids parameters. All the bond lengths were constrained with LINCS algorithm and the time step was set to 2fs. PME method with a cutoff of 1.0nm was used to treat the electrostatic interactions. The cutoff of nonbonded van der Waals (VDW) interactions was also 1.0nm. The system was first subjected to an energy minimization of 1000 MD steps, followed by a gradual heating to 300K. After that a 2 ns equilibrium run was performed with NPT ensemble at 1 atm and 300K. The final conformation of the 2ns run was utilized as the initial structure for further simulations. To test the stability of the native structure, we performed a conventional MD simulation of length 200 ns at 300K and 1atm with a NPT ensemble.

### Bias-exchange metadynamics

Metadynamics is a powerful sampling method that concentrates in the given collective variables (CVs) space and periodically applies repulsive Gaussian potentials on the CVs to accelerate the barrier-crossing events. Bias-exchange metadynamics (BEMD) further enhances the sampling efficiency by employing multiple replicas biased on different CVs and exchanging their configurations and velocities periodically according to a Metropolis-like criterion [31, 34, 35].

In our BEMD simulation, six replicas were used, where one replica was unbiased (the neutral replica) and the rest five were biased on different CVs, respectively. The CVs were chosen as the number of native hydrogen bonds (N_hb_) in the helix H_1_, the N_hb_ in the helix H_2_, the N_hb_ between H_1_ and the second loop L_2_, the radius of gyration (Rg) of the pseudoknot and the energy of the system. The height of Gaussian potential was set to 0.1 kJ/mol and the width was chosen as 0.3, 0.4, 0.25, 0.2nm and 100 kJ/mol for the five CVs described above, respectively. The time interval for depositing the Gaussian potentials was 1ps while the exchange attempting interval between replicas was 30ps. The simulation time of each replica was 500ns, resulting in a total of 3 microseconds. The program PLUMED (version 1.3) as a Gromacs plugin was used for the BEMD simulation [36].

### Free energy landscape (FEL) calculation and intermediates identification

Only the data from the neutral replica was used for further analysis. This is to avoid the problems that may exist in the biased replicas [37]. A detailed discussion of the reliability of the data from metadynamics can be found in our previous work [38]. The free energy landscape, taking a one-dimensional case for example, is calculated as

$$F(s)=-k_{B}T\text{ln}(P(s))$$ (1)

Where *s* is a desired CV, P(s) is the probability distribution along the CV, T is the simulation temperature and k_B_ is the Boltzmann constant.

From the FEL we identified the basins of attraction visually and then obtained their representative structures as follows. First the conformations were designated to different basins according to their CVs. Second, the conformations within the same basin were subjected to a clustering analysis. In brief, the *i-th* conformation was compared to each of the representative structures of the clusters obtained previously; if a RMSD smaller than a threshold was found, the *i-th* conformation was regarded as belonging to that cluster; otherwise, if the *i-th* conformation could not be assigned to any existing clusters, it was considered to be the representative structure of a new cluster. The representative structure of the largest cluster in a basin was used to represent the basin. The RMSD threshold in the clustering analysis was set to 0.1nm.

### Bound ions and ion-mediated structures

A "bound ion" is defined if a metal ion is within a distance of 0.4nm to any nucleotides. To characterize the effect of metal ions in mediating long-range interactions between different structural elements rather than a simple electrostatic screening effect, a “bridging ion” is defined if a metal ion simultaneously binds two or more nucleotides that are separated by at least 4 nucleotides along the sequence. If such an ion is detected in a structure, then this structure is defined as an “ion-mediated structure”. In a given structure ensemble, the ratio of the population of the ion-mediated structures over that of the non-ion-mediated structures is calculated to evaluate the importance of the mediating effect of cations in this ensemble. The equation reads,

$$R_{p}=\frac{P_{ion-mediate}}{P_{non-ion-mediate}}$$ (2)

If the value of R_P_ of an ensemble is greater than unity, the majority of the conformations therein will be ion-mediated structures, indicating that the mediating effect of metal ions plays a significant role in the stability of the ensemble.

## Results

### Stability of the native structure

The stability of the RNA pseudoknot is mostly attributed to three groups of interactions, including the canonical base-base pairing within two helices and the non-canonical interactions between loop L_2_ and H_1_ (Fig 1A and 1C). The last one is a tertiary interaction. The native structure is very stable under the current force field, according to the 200ns-long conventional MD simulation starting from the native structure. It shows that for most of the time, the fraction of the formed hydrogen bonds (HBs) exceeds 0.9 and the RMSD with respect to the starting structure is smaller than 0.4 nm (Fig 1 and S1 Fig). However, the tertiary interactions are comparably less stable, with a formation probability around 0.4 (Fig 1C).

It is commonly believed that metal ions play indispensable roles in RNA stability. In Fig 1B we show the structure that most represents the average cation binding pattern of the native state (S1 Fig). It can be seen that some cations are partly or deeply buried inside the RNA. For example, Na_1_^+^ binds to A8 and U28, and Na_2_^+^ interacts with both A8 and U11. By simultaneously interacting with nucleotides far away from each other along sequence, these metal ions bridge tertiary interactions and help stabilizing the RNA structure. Presumably, they play more active roles than simply acting as electrostatic screening agents.

### The free energy landscapes (FELs) and intermediates from the BEMD simulation

Based on the BEMD simulation data, we calculated the FEL on a two-dimensional surface defined by the number of HBs in H_1_ and that in H_2_ and monitored its change as a function of simulation time. It was found that the change was almost undetectable during the last 60ns of the 500ns BEMD run, suggesting a good quality of convergence (S2 Fig). Therefore the data collected until 500ns are used for further analysis.

**Fig 2:**
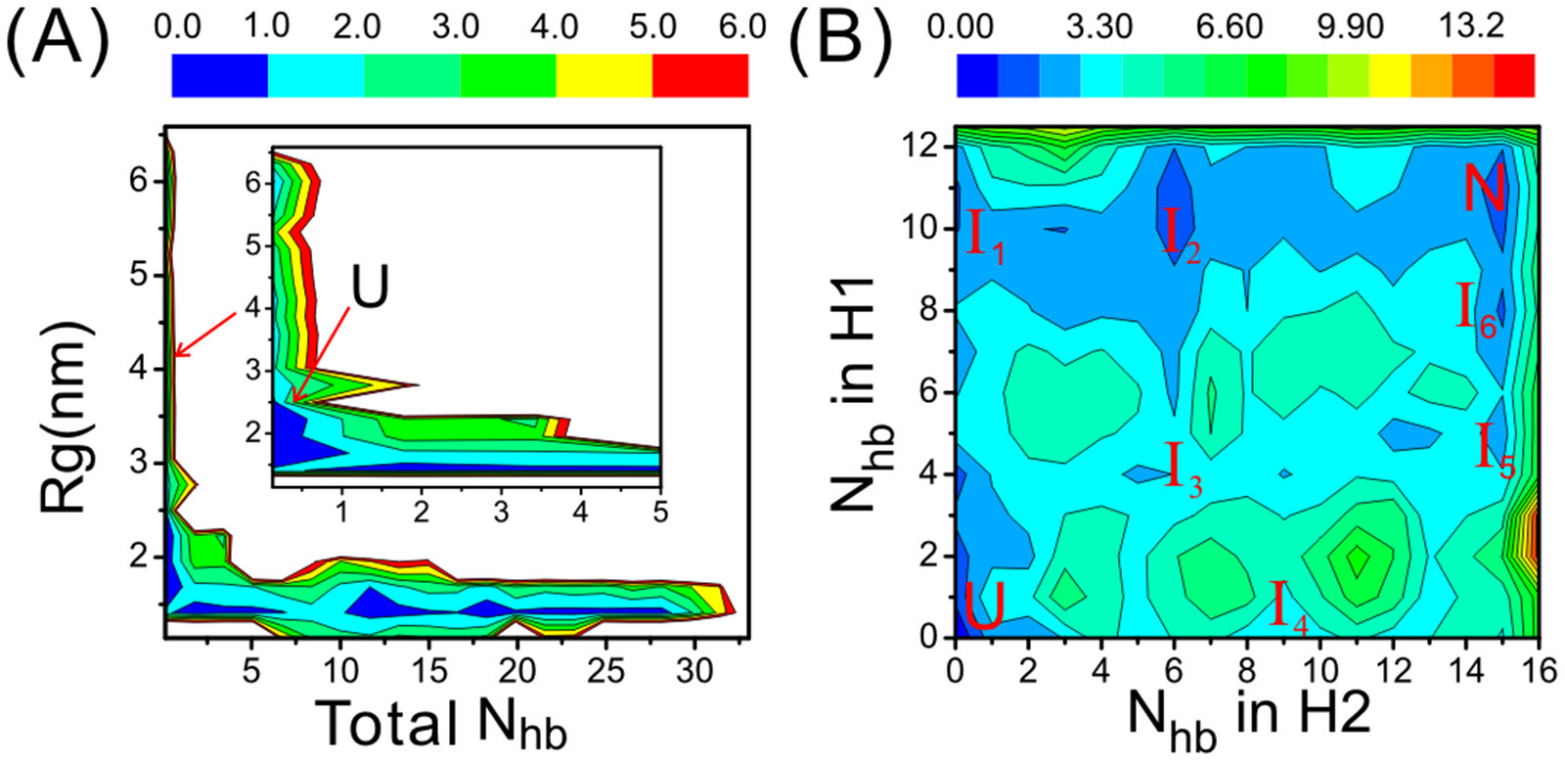
Free energy landscapes (FELs) calculated from the 500ns BEMD run. The magnitude of the free energy is represented by different colors, as quantified by the color scale on the top of the figure; the unit is kcal/mol. (A) The FEL projected on the radius of gyration (Rg) and the number of native hydrogen bonds (N_hb_). The inset is a zoom-in of the left region. (B) The FEL projected on the number of native hydrogen bonds in H_1_ and that in H_2_. The labels from I_1_ to I_6_, U and N denote six intermediate states, the unfolded basin and the native basin, respectively.

In Fig 2 we show two FELs calculated solely from the neutral replica of the BEMD simulation. The FEL projected on the collective variables (CVs) N_hb_ and Rg has an L-like shape, demonstrating that the RNA undergoes a structural collapse at its early folding stage, consistent with the previous findings [39]. Meanwhile, the FEL projected on the number of HBs in H_1_ and that in H_2_ reveals the existence of multiple basins of attraction, labeled as N, U, and I_1_~I_6_, respectively. Clearly, the basin-N and basin-U correspond to native states and unfolded states, respectively, supported by their positions in the FEL and structure analysis described in the following sections. The rest basins correspond to the folding intermediates and will be discussed later.

### Structure of the unfolded states

**Fig 3:**
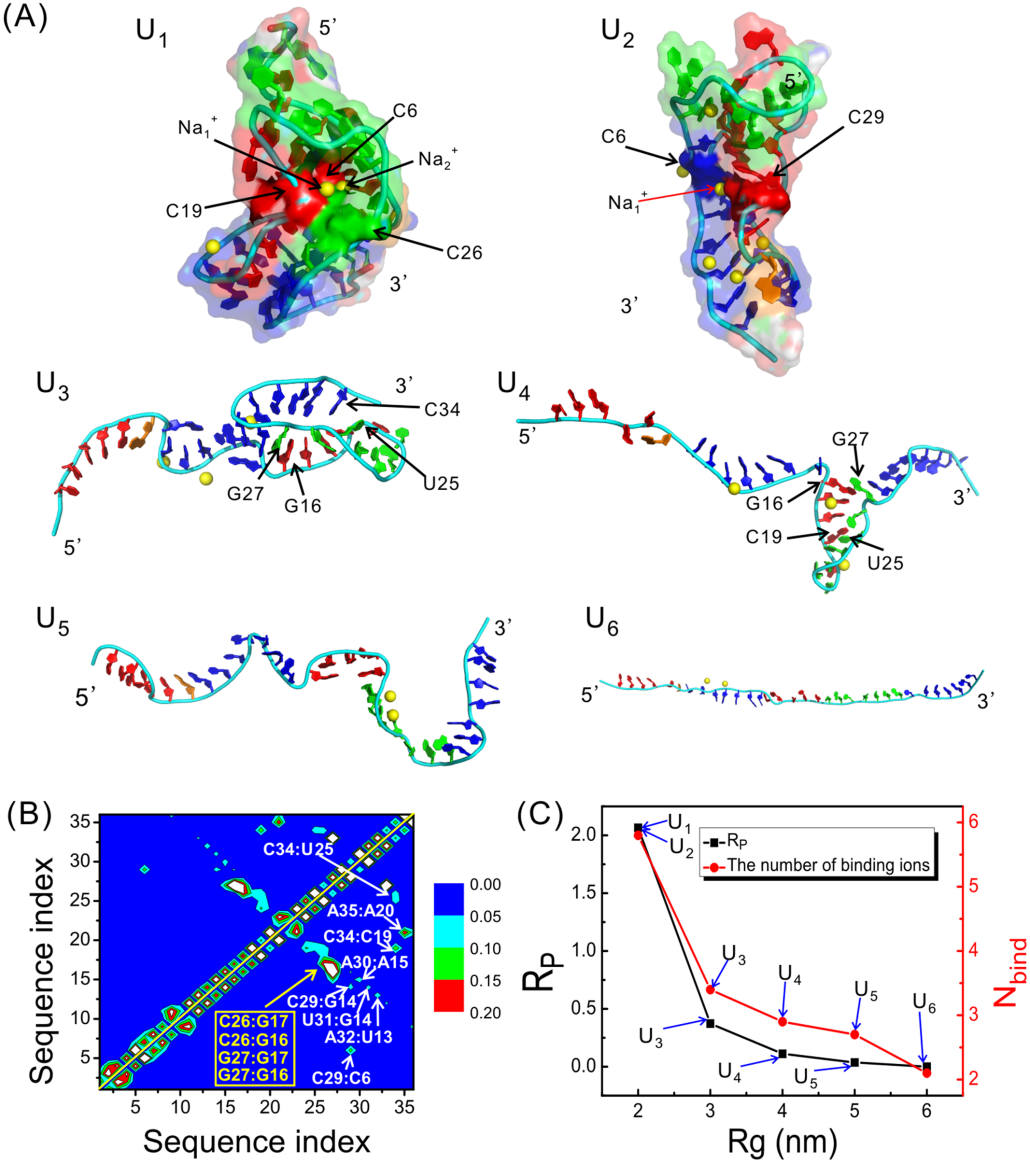
Structures of the unfolded states. (A) Representative structures of the largest six clusters in the unfolded states. (B) HB map averaged over all the structures in the unfolded states. The formation probabilities are indicated by different colors, as quantified by the color scale beside the figure. The labels inside the figure are also colored, with yellow indicating tertiary interactions between H_1_ and L_2_ and white non-native HBs. (C) The number of bound metal ions and R_p_ as a function of Rg plotted for the largest six clusters.

We performed cluster analysis on the denatured states. The largest six clusters are denoted from U_1_ to U_6_, respectively and shown in Fig 3. Their relative populations are about 26%, 18%, 8%, 5%, 5% and 4%, respectively. It can be seen that the unfolded states are structurally heterogeneous, with U_1_ and U_2_ compact while the other four partially or fully extended. At the first sight, the backbone of U_1_and U_2_ roughly resembles that of the native structure. However, a further calculation of the hydrogen bond map suggests that most hydrogen bonds are non-native; therefore both U_1_ and U_2_ are non-specific collapsed states. U_3_ is partially compact, with the 3'-end forming a triplex-like structure; however, most of the formed hydrogen bonds are still non-native, and its 5'-endis flipped out into solution. Compared with the previous structures, U_4_ is much more extended. It forms a hairpin between L_2_ and H_1_ through the base-pairs G16:G27 and C19:U25, which are, again, non-native. U_5_ and U_6_ are fully extended and no stable base-pairs are observed.

The formation probabilities of HBs averaged over all the conformations in the unfolded basin are shown in Fig 3B. It can be seen that most HBs are non-native. Interestingly, the tertiary HBs between L_2_ and H_1_ are also observed, for example, G27:G16 and G26:G17, although they are relative week even in the native structure.

Fig 3C shows the number of bound cations and the value of R_p_ as a function of Rg. It is clear that both of them increase rapidly as the molecule collapses. For the more extended states from U_3_ to U_6_, the average number of bound ions is less than 3 and R_p_ is less than 0.4. In contrast, for the compact states such as U_1_ and U_2_, R_p_ increases rapidly to about 2.0, indicating that the mediating effect of cations becomes more important in these structures. For example, in the structure of U_1_ shown in Fig 3A, Na_1_^+^ and Na_2_^+^ are trapped deeply inside the RNA and simultaneously bind to C6, C19 and C26, therefore drawing L_2_ and H_1_ close to each other. In U_2_ a similar role of metal ions is observed.

### Structures of the intermediates

**Fig 4:**
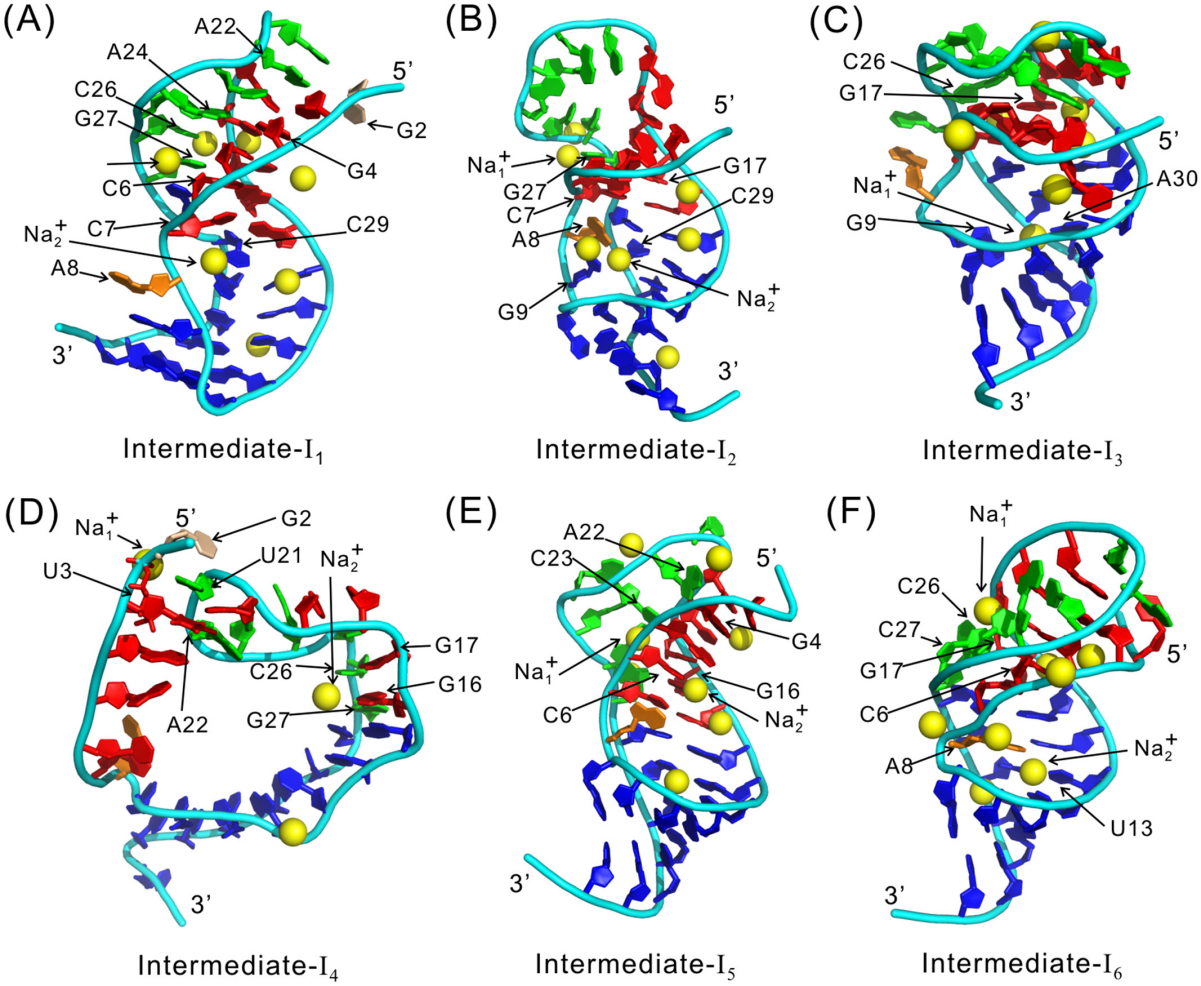
Representative structures of the intermediates. (A)-(F) are for the intermediates from I_1_ to I_6_, respectively. The nucleotides and metal ions are represented in the same way as in Fig 1.

**Fig 5:**
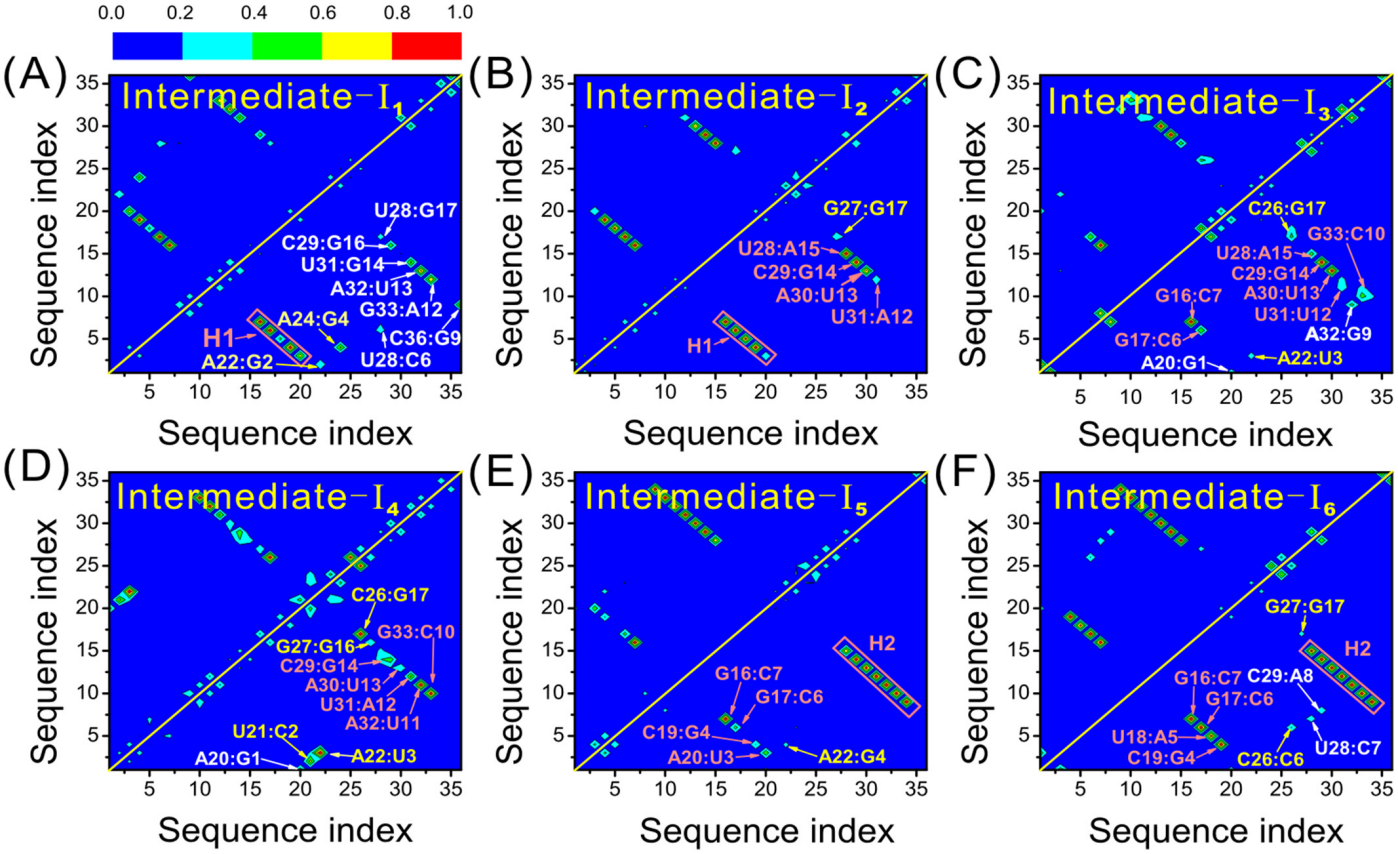
Average HB maps of the intermediates. (A)-(F) are for the intermediates from I_1_ to I_6_. Different colors of the HBs indicate different formation probabilities as quantified by the color sale on the top of the figure. The labels of the hydrogen bonds are also colored, with the red, white and yellow colors indicating the native, non-native, and tertiary HBs, respectively.

The representative structure of the intermediate-I_1_ is a triplex in which the helix H_1_ and the L_2_-H_1_ interaction have been formed and the nucleotides in the original H_2_ form a nonnative hairpin, as shown by Figs 4A and 5A. The L_2_-H_1_ interaction is established through A24:G4 and A22:G2. The Intermediate-I_2_ is different from I_1_ in that the nonnative hairpin is disrupted and the bases rearrange to form several native HBs (Fig 4B); at the same time the L_2_-H_1_ interactions in the triplex also rearrange to a new pattern. The intermediate-I_3_ is characterized by partly formed H_1_ and H_2_ (Figs 4C and 5C). The L_2_-H_1_ triplex has also formed, stabilized by A22:U3 and C26:G17. The intermediate-I_4_ is very different from I_1_, I_2_, and I_3_ in that H_2_ is almost formed while H_1_ not (Figs 4D and 5D); meanwhile, the two strands of H_1_, U3~C7 and G16~A20, interact with L_2_ through the tertiary contacts U3:A22, G17:C26 and G16:G27. The intermediate-I_5_ has a well formed H_2_ and a partly formed H_1_ (Figs 4E and 5E). In addition, the L_2_-H_1_ triplex is formed through A22:G4. The intermediate-I_6_ is a native-like state; most of the native base pairs have formed except A20:U3 in H_1_ (Figs 4F and 5F). In this intermediate, L_2_ and H_1_ interact with each other though C26:C6 and G27:G17.

**Fig 6:**
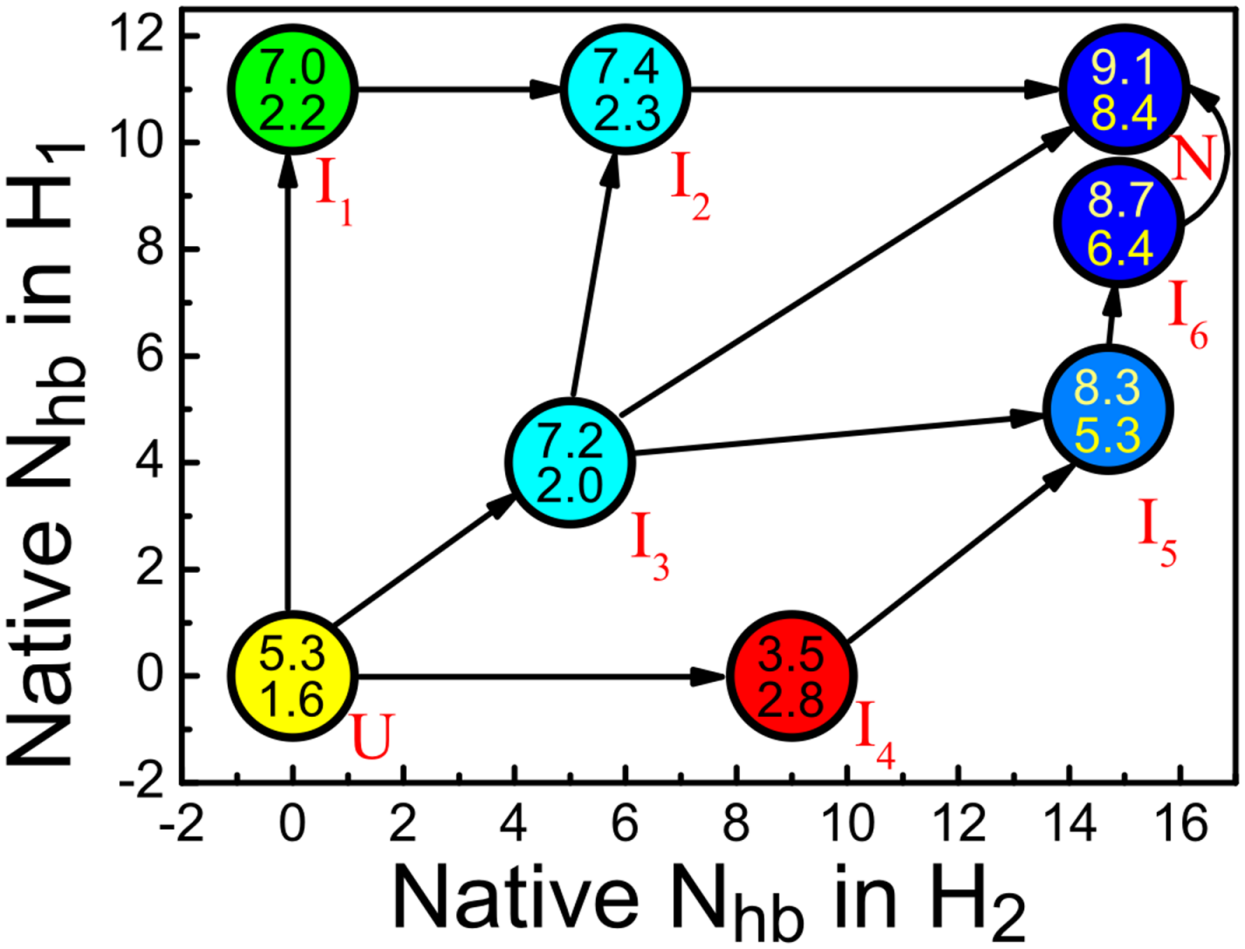
Binding of metal ions during folding process. The evolution of the number of bound metal ions (the upper number in the circle) and R_p_ (the lower number in the circle) during the folding process. The basins are depicted by circles and their positions roughly correspond to the CVs.

The evolution of the number of bound cations and R_p_ as folding proceeds through the above intermediates is given in Fig 6. It can be seen that R_p_ are larger than 2.0 for all the six intermediate states, implying that the mediating effect of metal ions are important for their stabilities. Take the intermediate I_1_ as an example, Na_1_^+^ binds to both C6 and C26 and pulls L_2_ and H_1_ together, hence stabilizing the triplex structure. Similar roles are observed for metal ions in the other intermediates, as can be seen from the representative structures in Fig 4. Moreover, Fig 6 shows that R_p_ increases as the molecule folds, indicating that when the structure becomes more similar to the native one, the mediating effect of cations becomes more important.

## Discussion

**Fig 7:**
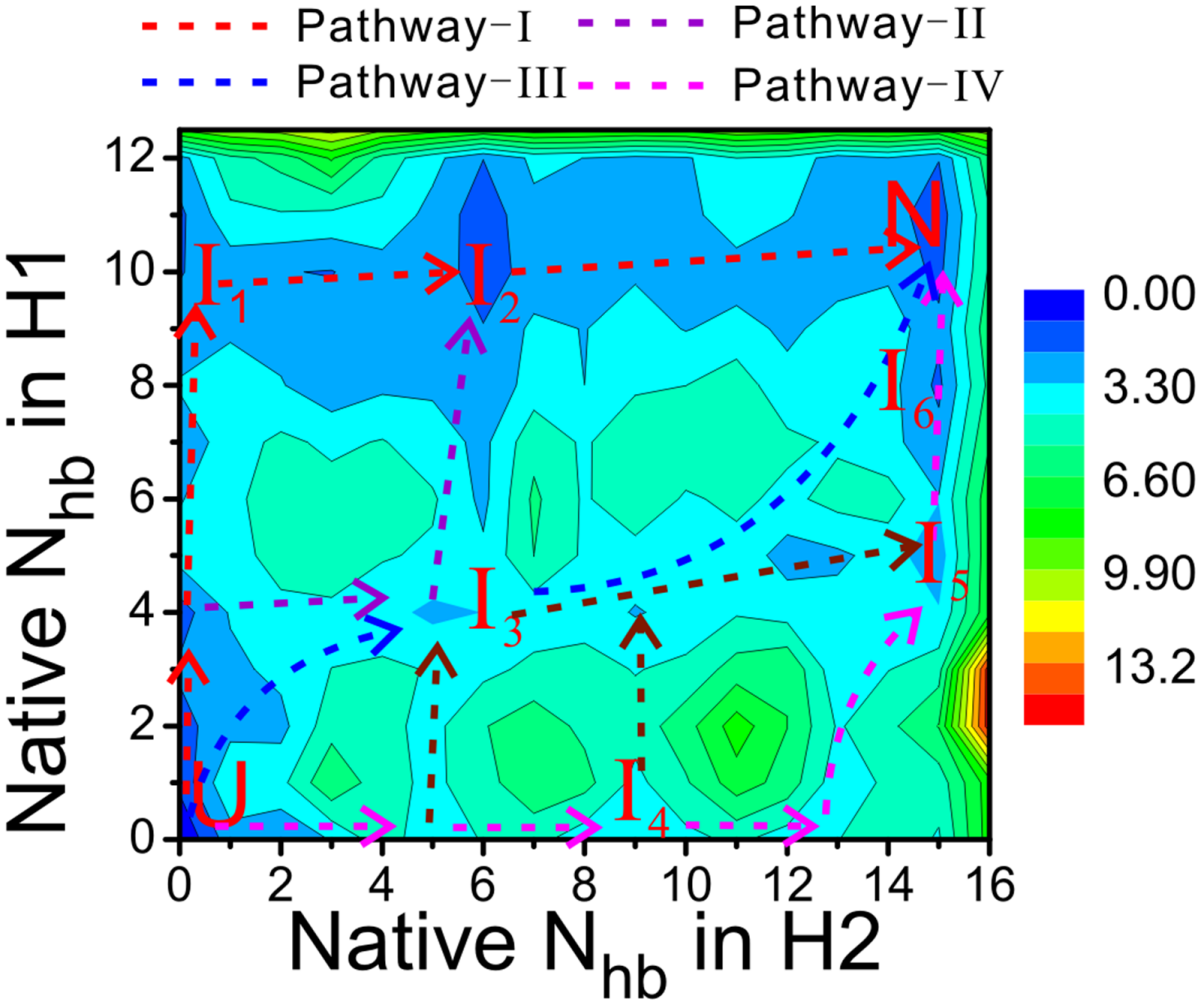
Multiple folding mechanisms proposed for the RNA pseudoknot. The typical pathways are labeled as pathway-I, pathway-II, pathway-III and pathway-IV, and are colored by red, violet, blue and wine, respectively.

The FEL obtained from our simulations shows multiple intermediates and hence suggests that the RNA pseudoknot folds via multiple mechanisms/pathways, summarized in Fig 7. Note that here the term pathway is used to describe the thermodynamic aspect of the FEL and does not necessarily reflect the underlying kinetics. The RNA may first form the helix H_1_ and then H_2_, or in an opposite order, corresponding to the pathway-I and pathway-IV, respectively. It may also form two helices simultaneously, corresponding to the pathway-III. The pathways-I and IV reflect a step-wise folding mechanism, i.e., one helix after the other, while the pathway-III corresponds to a cooperative mechanism. The folding may also proceed via a hybrid mechanism. For example, it may go via pathway-III to I_3_ first, and then to I_2_ or I_5_, from there it goes to the native basin via pathway-I or IV, respectively. Among all the pathways, the pathway-I is dominant since the relevant intermediates (I_1_ and I_2_) have lower free energy and larger entropy, estimated qualitatively from the size of the basins. The overall folding picture is consistent with the result for the same RNA obtained in our previous work [19], which suggested that the unfolding of the pseudoknot follows multiple pathway and the dominant one is a sequential unfolding of H_2_ and H_1_, corresponding to the pathway-I in this study. It worth mentioning that in our previous work the conclusion was drawn based on massive unfolding MD simulations and therefore reflected the kinetic nature of the underlying FEL. In contrast, the BEMD simulation in this work sacrifices the kinetics and concentrates on the thermodynamic aspect of the FEL. The consistence between FELs and pathways drawn from two very different approaches is amazing and bolsters the presented findings.

Multiple pathway mechanism has been frequently observed for RNAs in experiments. For example, Chen et al. studied the mechanical folding and unfolding of an H-type pseudoknot in hTR RNA with optical tweezers and found that at low forces the folding occurs step by step, with 5'-stem first followed by 3'-stem, in contrast to one-step unfolding at high forces of ~46pN [9]. This is direct experiment evidence that the folding of pseudoknot takes complex pathways, according to the authors. Also using optical tweezers, Wu et al. studied the structural dynamics and rearrangements of the *rpsO* operator RNA and suggested that the molecule folds to the high-stability-pseudoknot via a double-hairpin structure, or via a low stability pseudoknot as an intermediate [22]. The double-hairpin pathway is reminiscent of the pathway-III in this study. Messieres et al. investigated the unfolding pathways of a -1 PRF sequence in CCR5 mRNA with optical trapping techniques; they found the RNA manifests several distinct unfolding pathways when subject to end-to-end force [40]. In fact, even for a simple RNA hairpin, large-scale simulations revealed multiple intermediates and suggested that folding can start either from the closing base pair or from the end base pair [41].

Multiple folding pathways of RNAs and the underlying mechanism have been discussed by Cho et al, who studied the folding of three H-type RNA pseudoknots based on a structure-based coarse-grained model [14]. They found that the pseudoknots fold through hierarchical, cooperative mechanism, or via multiple pathways. They further proposed that the folding order is mostly determined by the stabilities of the isolated secondary structure. Following this suggestion, we calculated the stabilities of H_1_ and H_2_ based on Turner’s free-energy rule [42] and found values of -10 and -12.4kcal/mol, respectively. The slightly stronger stability of H_2_ seems to indicate a first folding of H_2_ and then H_1_, contrary to the observation in our simulations. However, we notice that the tertiary interaction between H_1_ and L_2_ plays an indispensable role; according to our simulation, the tertiary HBs in this region are always observed in the intermediates I_1_, I_2_ and I_3_ and they essentially make the RNA a triplex. Therefore, it is hypothesized that the tertiary interactions, although rather weak themselves, stabilize the helix H_1_ and bias the folding flux to the pathway-I. Therefore the stability rule of folding order proposed by Cho et al. still holds but needs to consider the contribution from tertiary contacts. Interestingly, in the intermediate I_4_, the tertiary interactions form earlier than H_1_ and are particularly important for maintaining the overall structure. Presumably, they will also affect the folding flux through the pathway-IV. Similar role of tertiary interactions has been discussed by Cho et al [14]. They found that for the hTR pseudoknot, the tertiary contacts can form before the complete assembly of secondary structures, suggesting that they are significant in determining the folding cooperativity and pathways in some cases. An experimental study of the folding bacterial group I ribozyme also demonstrated that the tertiary interactions between helices bias the structural ensemble toward native-like conformations and therefore are important for determining the folding process.

It is of particular interest to discuss the folding rates of RNA pseudoknots. Experimentally, the measured folding rates vary greatly. In the optical tweezer study of the hTR pseudoknot, Chen et al. observed two-state folding transitions at ~10 and ~5 pN with ensemble rate constants of ~0.1 sec^-1^; an extrapolation to zero force yields an apparent time constant of ~60 ms[9]. In a T-jump perturbation experiment for VPK, a variant of the MMTV pseudoknot designed to avoid kinetic traps, Narayanan et al. measured folding times of 1–6 ms, which are at least 100-fold faster than previous observations of very slow folding pseudoknots that were trapped in misfolded conformations [18]. Green et al. studied the folding/unfolding kinetics of an H-type pseudoknot from IBV with optical tweezers over a force range of 15–20 pN; the apparent folding times extrapolated to zero force is ~1.8 microsecond [25]. We also estimated the transition times between basins for the gene32 mRNA pseudoknot by constructing a Markov state model based on the calculated FEL, following the procedure used by Marinelli and colleagues [35] (S1 Text and S5–S7 Figs). It is found that the transition times are mostly of the order of microseconds, and there are no obvious kinetic traps that can hold the RNA for a long time (S7 Fig). An interesting discovery is that, despite of the existence of multiple intermediates and folding pathways, the ensemble folding kinetics is single-exponential, fitted from the time dependence of the fraction of the folded trajectories (S6 Fig). The fitted rate is determined to ~23μs^-1^, two orders of magnitude faster than that of VPK [18] but ten times slower than that of the pseudoknot from IBV [25]. Since direct experimental measurement of the folding rate of this RNA is currently lacking, we cannot make concrete conclusions regarding this issue. However, we do not expect that the RNA will fold at such a fast rate in experiments, based on the following reasons. First, the pre-defined CV space and its latter coarse-graining process in the BEMD simulation may conceal some kinetic traps. Second, the transition times were calculated based on the assumption that the diffusion constant is independent on the position on FEL, which is a very rough approximation and may actually fail for this RNA. Therefore, the folding rate estimated here may represent an upper-limit and gives a hint of how fast this RNA may ultimately fold, if potential traps, if exist, can be avoided by a careful design of experiments. It is interesting to wait experimentalists to measure the actual folding rates of the RNA and make comparisons.

It is worth discussing the role of non-native interactions in the folding process. In the unfolded states, the majority of HBs are non-native while native HBs are hardly seen in our simulation. This feature is in contrast to some cases in protein folding, where a significant amount of native contact can be observed in the denatured states, such as in protein villin headpiece [43, 44]. Whether this feature is unique to the specific RNA studied here or a general principle is not known and the answer needs a significant amount of future work. Non-native interactions are also frequently observed in the early folding intermediates such as I_1_. Apparently, these non-native interactions have to break for the RNA to fold to the next stage and hence essentially hinder the folding process. It is interesting to mention that the opposite role of non-native interactions has been observed in other cases, such as in the folding process of a DNA quadruplex [45] and an intrinsically disordered protein inhibitor IA3 [46], where non-native interactions were found to be able to facilitate the folding process by reducing the searching phase space.

Metal ions are crucial for the initial collapse of the denatured state as well as the later folding stages. According to our simulation, even in the unfolded basin, the correlation between the structural collapse and the number of bound ions is obvious (Fig 3A). This correlation also holds for the later folding process to the native state via several intermediates, the correlation between folding and binding (Fig 6). To be more specific, inside the unfolded state the number of bound cations increases from 2 for the extended structures to about 6 for the most compact structures; and the average increases from 5.3 for the unfolded state to 9.1 for the native state. The correlation shows that the binding of cations is indispensable for the folding process of the RNA. In addition to this correlation, there is also a correlation between folding and the value of R_p_, which measures the significance of cations in bridging long-range interactions. According to Fig 6, R_p_ increases from 1.6 for the unfolded state to 8.4 for the native state. The continuous increase of R_p_ suggests that the mediating effect of cations becomes increasingly important during folding. This picture is in agreement with that given by previous studies [15, 47, 48], where metal ions were shown to be essential partners for the folding of RNAs, playing the roles of neutralizing the negatively charged backbone as well as mediating the formation of intermediate structures.

Here we want to mention that the bound ions in our simulations may correspond to the tightly bound ions proposed in the TBI theory [30, 49]. The benefit given by our simulation is the atomistic binding information of metal ions on the intermediates, which may be combined with such models and give a better description of the cation-RNA interactions. Such models are important since it has been shown that the intermediates have to be considered for the effect of cations on RNA stability [27, 50].

Caution should be given regarding the accuracy of the force field. In a recent study of the folding of RNA tetraloops, Chen and Garcia suggested that the AMBER99 force field for nucleic acids results in bloated nucleobases that do not accurately reflect the physiochemical properties of aqueous heterocycles [51]. Here we argue that this deficiency may not seriously affect the folding mechanism, since the latter is mostly determined by the balance between enthalpy and entropy in searching for the native basin of attraction. In fact, a previous work from the same group [52] studied the pressure and temperature folding/unfolding equilibrium of a small RNA hairpin with replica exchange molecular dynamics simulations and found that the AMBER99 force field is able to fold the molecule from extended conformation to structures with RMSD within 0.4–0.6 nm to the crystal structure. However, a quantitative estimation of the influence of this deficiency on the folding mechanism obtained here is not available yet, which needs substantial further works.

In summary, in this work we studied the free energy landscape of an H-type RNA pseudoknot using an advanced sampling technique and all-atom molecular dynamics simulation with both RNA and water explicitly modeled. Multiple intermediates were detected and their structures were analyzed. It is suggested that the folding follows multiple mechanisms, including a step-wise mechanism starting either from the first helix or the second, and a cooperative mechanism with both helices forming simultaneously. Despite of the existence of multiple intermediates and pathways, the ensemble folding kinetics estimated from a Markov state model is single-exponential, with a folding rate of ~23μs^-1^. This value may represent an upper-limit and gives a hint of how fast this RNA may ultimately fold, if potential traps can be avoided. The roles of metal ions are also analyzed. It is shown that the correlation between folding and binding is significant, and the bound ions mediate long-range interactions, stabilizing the intermediate structures. We believe this study represents a step forward in understanding the folding process of RNA pseudoknots.

## Supporting Information

S1 Fig Results from the MD simulation for the native structure. The simulation was started from the native structure and lasted for 200ns. It was designed to test the stability of the native structure under the current force field. The details are described in the main text. (A) The distribution of RMSD of the structures collected from the trajectory. (B) The RMSF of each nucleotide calculated from the trajectory. (C) The Na+ ion binding probabilities of the nucleotides. The labels in the x-axis are the sequence indices of the RNA nucleotides.
(TIF)

S2 Fig Convergence test for the BEMD run. (A) The FELs for the first 400ns simulation. (B) The FELs calculated from the last 60ns.
(TIF)

S3 Fig The relative population of the largest 20 clusters in the unfolded states. (TIF)

S4 Fig The Na^+^ ion binding probabilities for the nucleotides of the six intermediates. (TIF)

S5 Fig The diffusion constant as a function of the lag time. The results were calculated from the 200ns MD for the native structure.
(TIF)

S6 Fig The ensemble folding kinetics calculated from KMC simulations. The black curve is the raw data and the red one is from a single-exponential fitting.
(TIF)

S7 Fig The transition times between different basins. The results were estimated from the Markov state model and kinetic Monte Carlo simulations.
(TIF)

S1 Text The construction of Markov state model. (PDF)
