# Supplementary figures and images for: Free Energy Landscape and Multiple Folding Pathways of an H-Type RNA Pseudoknot
Source: PLoS One. 2015 Jun 1;10(6):e0129089. doi: 10.1371/journal.pone.0129089 (PMC4451515; doi:10.1371/journal.pone.0129089)

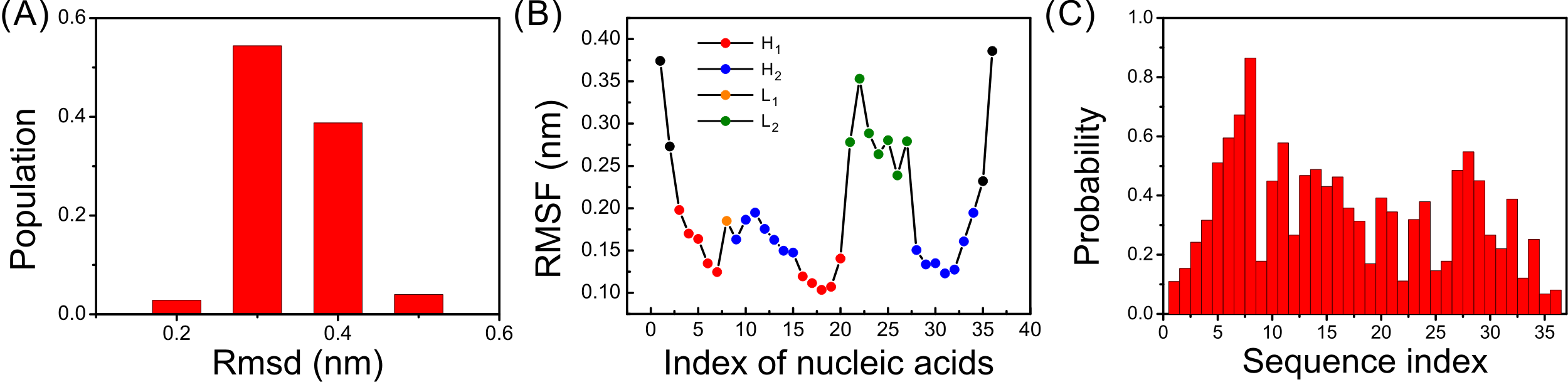

Supplement: S1 Fig — The simulation was started from the native structure and lasted for 200ns. It was designed to test the stability of the native structure under the current force field. The details are described in the main text. (A) The distribution of RMSD of the structures collected from the trajectory. (B) The RMSF of each nucleotide calculated from the trajectory. (C) The Na+ ion binding probabilities of the nucleotides. The labels in the x-axis are the sequence indices of the RNA nucleotides. (TIF) [file pone.0129089.s001.tif]

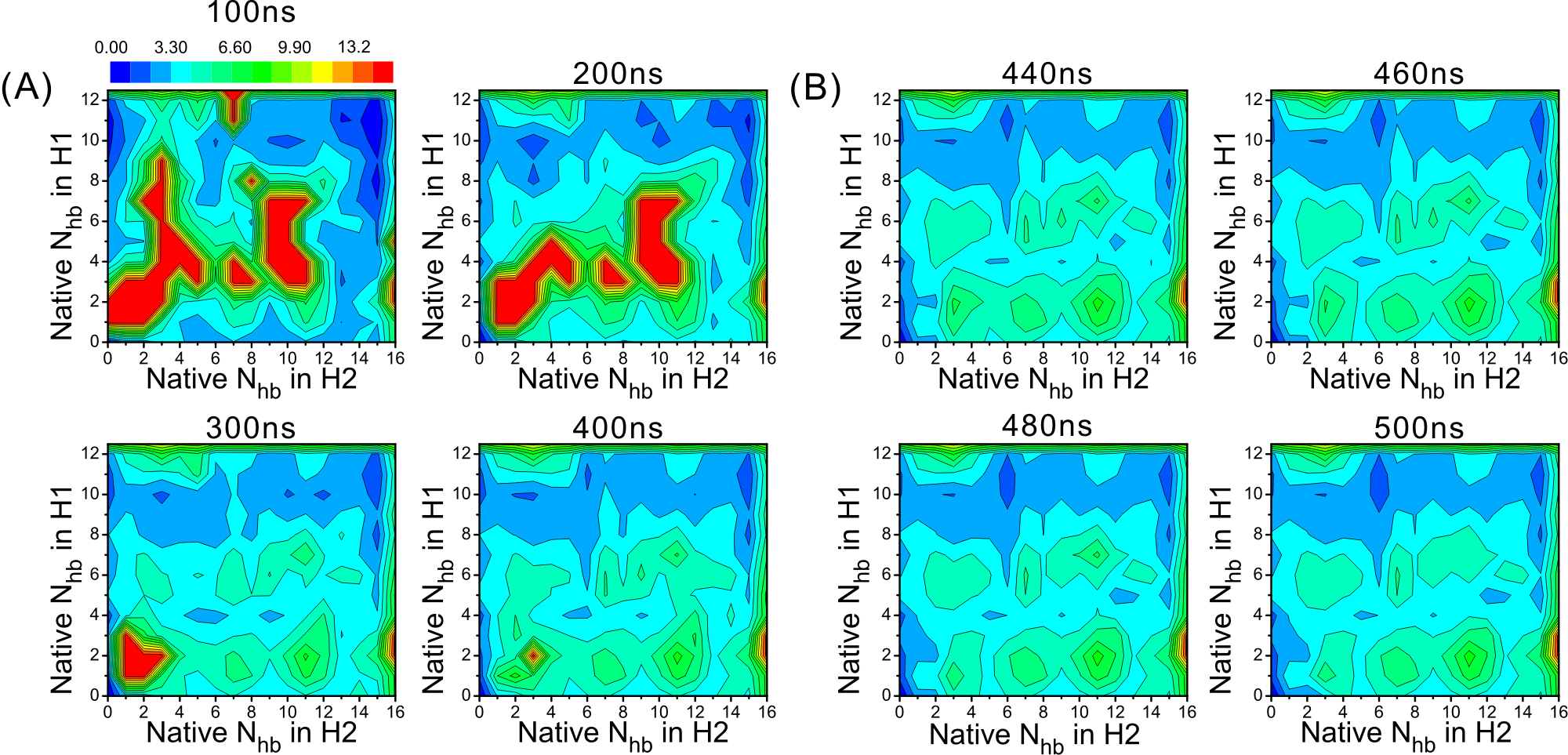

Supplement: S2 Fig — (A) The FELs for the first 400ns simulation. (B) The FELs calculated from the last 60ns. (TIF) [file pone.0129089.s002.tif]

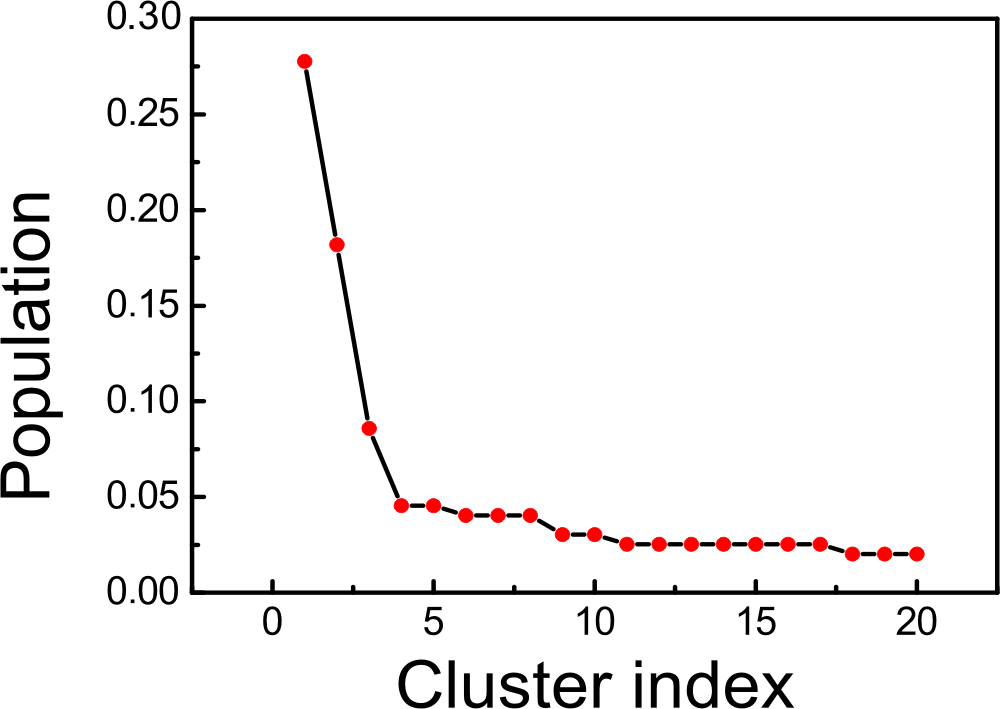

Supplement: S3 Fig — (TIF) [file pone.0129089.s003.tif]

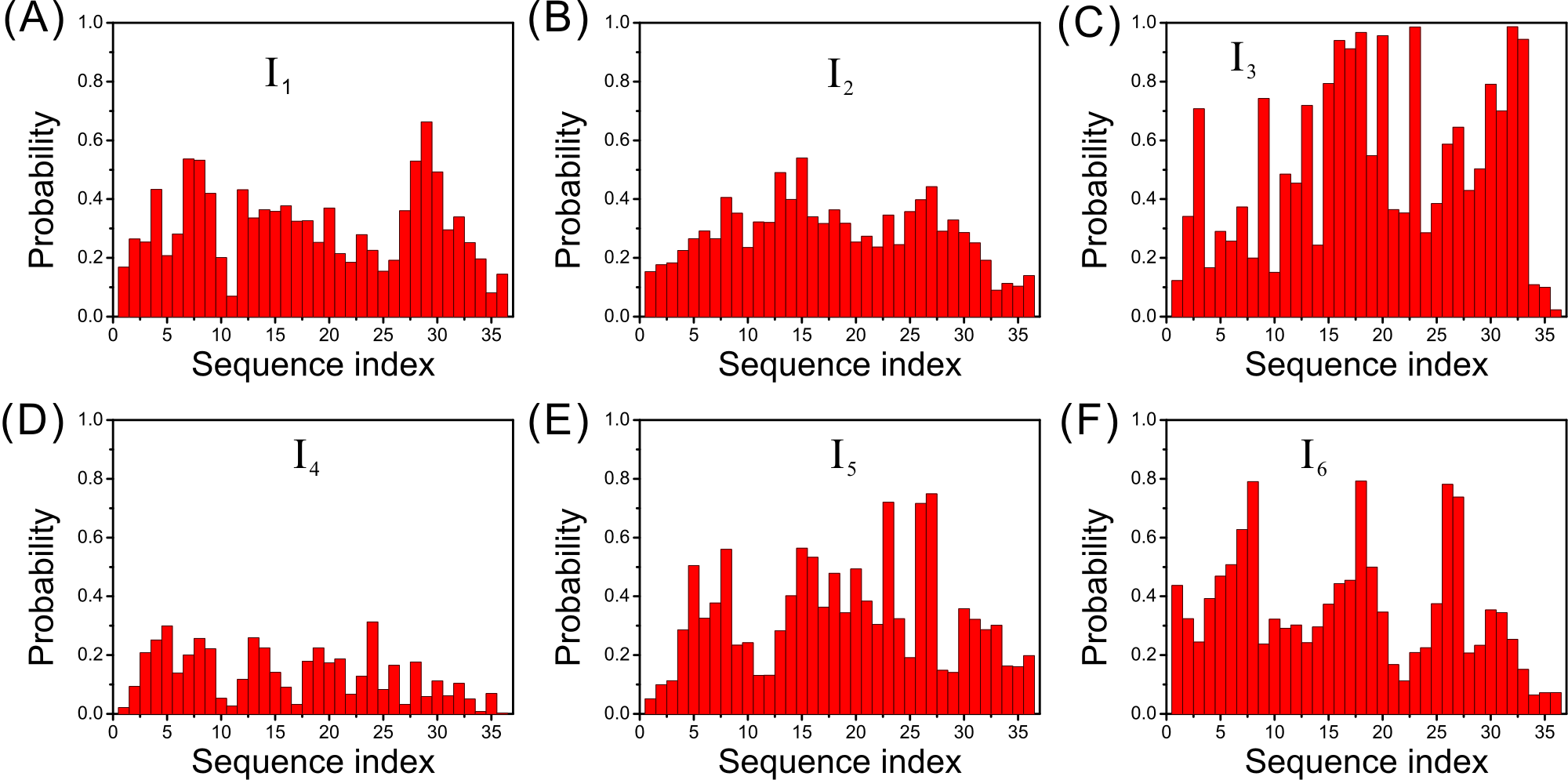

Supplement: S4 Fig — (TIF) [file pone.0129089.s004.tif]

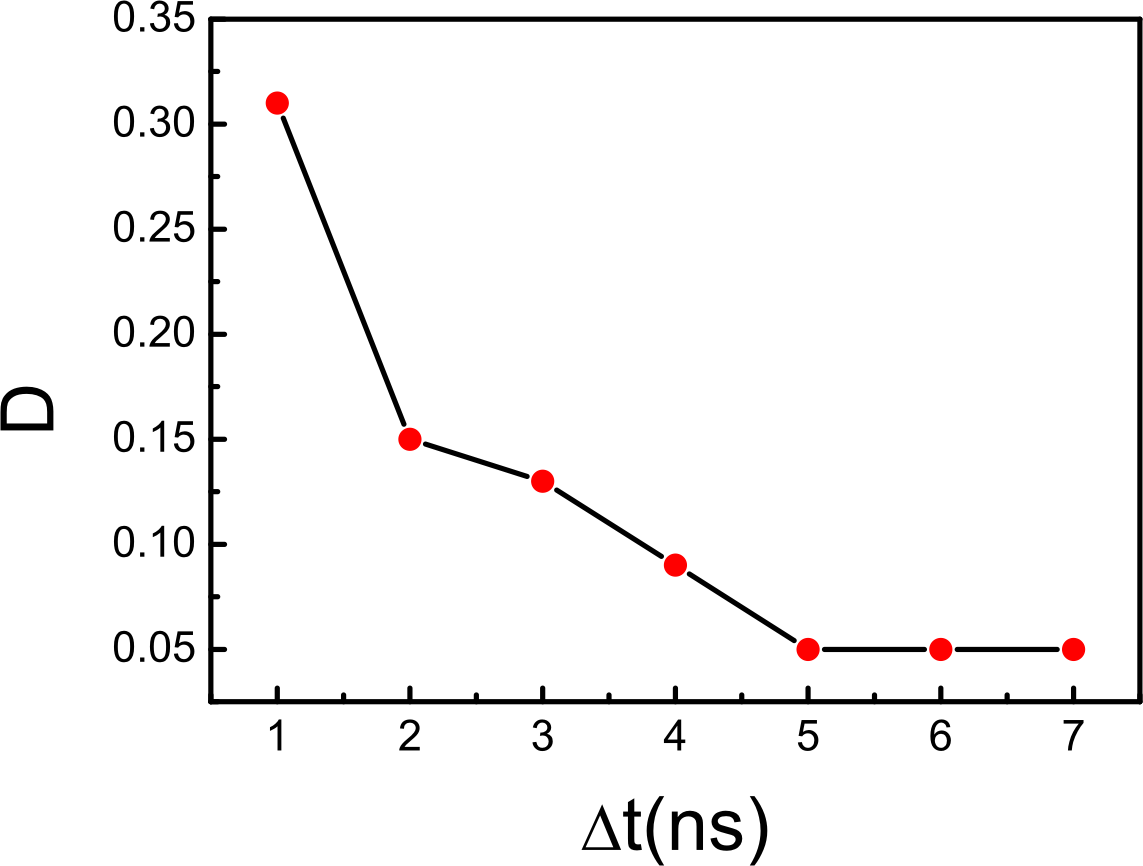

Supplement: S5 Fig — The results were calculated from the 200ns MD for the native structure. (TIF) [file pone.0129089.s005.tif]

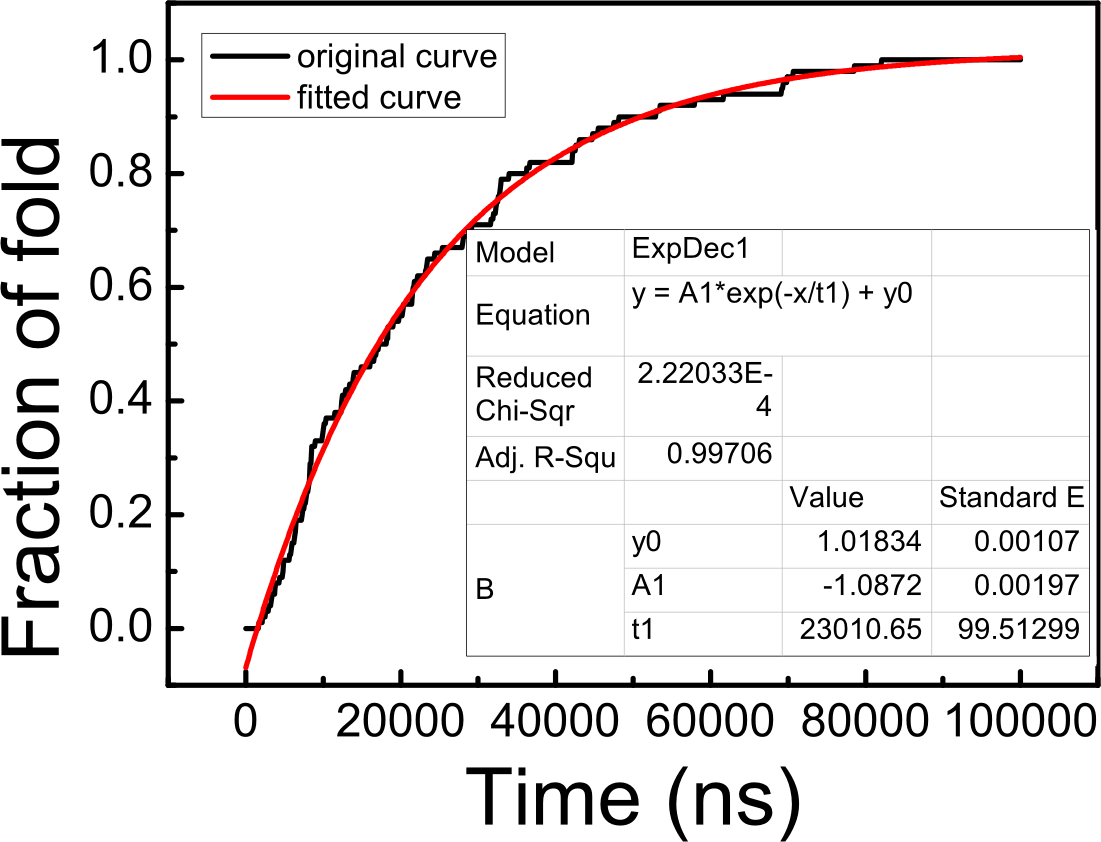

Supplement: S6 Fig — The black curve is the raw data and the red one is from a single-exponential fitting. (TIF) [file pone.0129089.s006.tif]

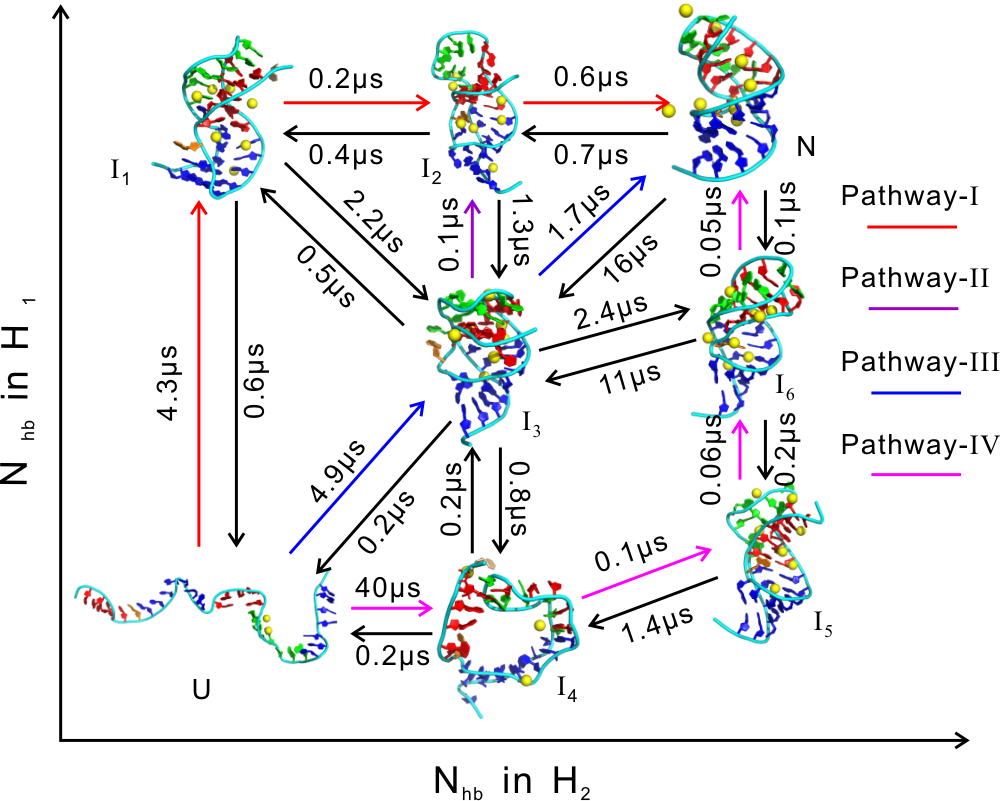

Supplement: S7 Fig — The results were estimated from the Markov state model and kinetic Monte Carlo simulations. (TIF) [file pone.0129089.s007.tif]
