# Supplementary material for: Free Energy Landscape and Multiple Folding Pathways of an H-Type RNA Pseudoknot
Source: PLoS One. 2015 Jun 1;10(6):e0129089. doi: 10.1371/journal.pone.0129089 (PMC4451515; doi:10.1371/journal.pone.0129089)
Supplement: S1 Text — (PDF) [file pone.0129089.s008.pdf]

S1 Text. The construction of Markov state model

The Markov state model

To obtain folding kinetics from the simulation, we constructed a Markov state model following the procedure used by Marinelli and colleagues [1]. We first partitioned the free energy landscape into mesoscopic states (bins). The term “mesoscopic” emphasizes that the bins have to be small enough to lower the probability of grouping kinetically irrelevant microscopic states in one state and have to be simultaneously large enough to guarantee sufficient statistical quality. The transition rates between bins were assumed to be

$$k_{ij} = k_{ij}^0 \exp(-\beta \Delta F_{ij} / 2) \qquad (1)$$

where *i* and *j* are the indices of the bins, k_{ij}^0 are the rates associated with simple diffusion on a flat free energy surface, ΔF_{ij} are the free energy difference between bins *I* and *j*, and β is the inverse temperature. The rates k_{ij}^0 are proportional to the diffusion constants *D* on the free energy surface and taken to be position independent for simplicity. The way we determined the diffusion constant is described in the next section.

The transitions between Markov states can be described by a stochastic matrix T, which is related to the rate matrix K=(k_{ij}) by

$$T(\Delta t) = e^{K\Delta t} \qquad (2)$$

where Δt is the time lag between two successive observations of the system states.

Estimation of the diffusion constants

The diffusion constant was determined by maximizing the likelihood that a given MD trajectory is generated by the corresponding Markov state model. The likelihood L(D) as a function of D is given by

$$\ln L(D) = \prod_t p_D(\alpha(t + \Delta t) \mid \alpha(t)) \qquad (3)$$

where $\alpha(t)$ is the index of the bin at time t for a given MD trajectory. The trajectory was taken from 200ns long MD run starting from native structures. Note that the likelihood L(D) is also a function of the time lag Δt, thus the diffusion constant also depends on this time lag. A common behavior is that by increasing the time lag the diffusion constant converges to a well defined value. This means that after this Δt, the dynamics between bins is close to Markovian and is well approximated by the model proposed. The logarithm of the likelihood $\ln L(D)$ as a function of D, and the dependence of D on Δt are given in Fig. S5(A).

The transition times between different clusters

Kinetic Monte Carlo (KMC) method [2] was employed to estimate transition rates between clusters. The transition rate from cluster A to B was calculated by monitoring the number of times a trajectory goes from A to B without passing any other basins as a function of time during a long kinetic Monte Carlo simulation. In principle, this number grows linearly with time *t*,

$$N_{BA} = k_{BA} P_A t \qquad (4)$$

where P_A is the population in basin A. k_{BA} can be calculated by dividing the slope of the curve N_{BA}

as a function of t by P_A. The slope was obtained by a linear fit of the curve. The transition time was then given by

$$\tau_{BA} = 1 / k_{BA} \qquad (5)$$

The overall folding kinetics

To gain knowledge of the ensemble folding kinetics, we made a calculation of the fraction of the folded RNA as a function of time with KMC simulations. To this end, we started hundreds of KMC simulations from the unfolded states, and recorded the number of trajectories that reached the native states every 100ns. Finally, we fit the fraction of the folded as a function of time with a single exponential function, as shown in Figure S5(B). Double-exponential fitting was also performed but gave two relaxation time constants of the same, indicating that a single-exponential fitting is already sufficient. Therefore the figure for double-exponential fitting is not presented.

References

1. Marinelli F, Pietrucci F, Laio A, Piana S (2009) A Kinetic Model of Trp-Cage Folding from Multiple Biased Molecular Dynamics Simulations. PLoS Comput. Biol. 5: e1000452.
2. Bortz AB, Kalos MH, Lebowitz JL (1975) A new algorithm for Monte Carlo simulation of Ising spin systems. *J. Comput. Phys.* 17: 10-18.
